# The use of mobile phones for demographic surveillance of mobile pastoralists and their animals in Chad: proof of principle

**DOI:** 10.3402/gha.v7.23209

**Published:** 2014-02-04

**Authors:** Vreni Jean-Richard, Lisa Crump, Doumagoum Moto Daugla, Jan Hattendorf, Esther Schelling, Jakob Zinsstag

**Affiliations:** 1 Swiss Tropical and Public Health Institute (Swiss TPH), Basel, Switzerland; 2 University of Basel, Basel, Switzerland; 3 Centre de Support en Santé Internationale (CSSI), N'Djaména, Chad

**Keywords:** mobile phones, mobile pastoralists, demographic surveillance, herd surveillance, one health

## Abstract

**Background:**

Demographic information is foundational for the planning and management of social programmes, in particular health services. The existing INDEPTH network surveillance sites are limited to coverage of sedentary populations. Including mobile populations in this approach would be expensive, time consuming and possibly low in accuracy. Very little is known about the demography of mobile pastoralists and their animals, so innovative approaches are urgently needed.

**Objective:**

To test and evaluate a mobile demographic surveillance system for mobile pastoralist households, including livestock herds, using mobile phones.

**Design:**

Mobile pastoralist camps were monitored (10 for 12 months and 10 for 18 months) using biweekly mobile phone calls with camp leaders and their wives to conduct interviews about the households and livestock. The collected information was validated through personal visits, GPS data and a livestock demographic model.

**Results:**

The study showed the feasibility of mobile phone surveillance for mobile pastoralist camps, providing usable, valid information on human and livestock population structures, pregnancy outcomes and herd dynamics, as well as migration patterns. The approach was low-cost and applicable with the existing local resources.

**Conclusion:**

Demographic surveillance in mobile populations is feasible using mobile phones. Expansion of the small-scale system into a full mobile demographic surveillance system is warranted and would likely lead to improved planning and provision of human and animal health care.

Demographic information is essential to expand concepts of social development and to plan land management and social programmes, in particular health services. Without demographic data, it is challenging to accurately assess the effectiveness of interventions (1).

To address the lack of national demographic information systems in developing countries, the international INDEPTH network has developed demographic and health surveillance systems in many locations around the world. In 43 sites in 20 countries, a population of 3.2 million people is collectively followed with regular visits to update demographic parameters (2). However, these surveillance sites have been limited to coverage of sedentary communities. Very little is known about the demography of mobile populations such as working migrants or mobile pastoralists and their herds. Only a handful of studies with demographic indices in African mobile pastoralists are available (3–8). In Chad, one of the poorest countries of the world, estimates of the mobile pastoralist population range from less than 400,000, or 3.5% of the total population in the 2009 national census, up to 2 million (9, 10). Despite the low numbers, these populations are reported to own about half of the livestock and generate about half of the national meat production (10), representing great economic potential. Although several authors have previously described the mobile pastoralist populations in the Lake Chad area (1, 11), longitudinal data on demographic development of the human and animal populations is lacking (12, 13).

For health services, existing local systems are not conceptualised to include mobile populations, for example, to overcome the obstacles of cultural and language-related access problems (14, 15). Additionally, mobile communities face discrimination in health centres (16). An earlier study showed that livestock had much better vaccination coverage than did children less than 5 years of age, none of whom had been completely immunised according to the recommended programme. Subsequently, a programme of simultaneous vaccination for animals and children was introduced and sustained in the area for 5 years, resulting in a much higher vaccination coverage rate in children (17–19).

There is clearly a need for adapted, integrated services as well as for the establishment of demographic baseline data. Demographic surveillance as developed for sedentary communities is not feasible with mobile pastoralists, because the regular visits need to take place in different locations depending on migration routes. This is costly and time consuming, involving enormous logistical efforts. However, without such basic information, the health services cannot adequately plan to include pastoralists in either fixed or outreach services.

Demographic surveys for African mobile populations have mainly been conducted to generate information on fertility and mortality rates (20) rather than to estimate population sizes and densities. In demographic population surveys or censuses, mobile pastoralists, or even entire regions where they are a majority, were often excluded (21). Alternative methods have been used previously, for example, the ‘water-point approach’ where data is collected at the water bodies and wells used by mobile pastoralists, but these are limited by high personnel costs and logistical issues (16). Another approach is aerial censuring, which requires validation for people and complementation with field data, e.g. for household structures (22). A significant shortcoming of this approach for the Lake Chad area is that people and animals often shelter under trees during hot periods of the day. Weibel et al. tested an adaptation of the population ecology capture–mark–recapture method to estimate the population size in the south-eastern Lake Chad area using biometric fingerprinting and random transects (1). Although this method proved to be feasible, the recapture probability was too low for an accurate estimation in the large population that spread over several countries.

The benefits of using mobile phones for health information and interventions have been shown in developing country settings, using text messages (23) and phone calls (24, 25). The benefits of mobile phone use have additionally been described in animal health and disease control (26, 27). In the last decade, mobile phone communication increased rapidly in Africa, to 45% penetration across the continent (28). While one in three Chadians owned a mobile phone by the end of 2011 (29), penetration rates in Chad are reported to be well below the African average, with patchy network coverage particularly in rural areas (30). Most of the mobile pastoralist camps (consisting of an extended family group) in our study area had at least one household that owned a mobile telephone (personal observation). It is expected that coverage will steadily increase and this will be associated with internet availability.

Since demography of mobile settlements includes many locations due to the movement of communities, it is also important to collect data about the transhumance routes. Data in Gorane and Arab communities in the area were collected by Wiese in 1998 and 1999 using participatory mapping (31), but the reporting was not in real time.

The objective of this study was to test and evaluate a new method for demographic surveillance of mobile communities and their livestock using mobile phones. We call this a mobile demographic surveillance system (mobile DSS) because the covered populations have, at least partially, a mobile lifestyle. We combined animal and human demographic surveillance because of the close interactions of the study population with livestock, because of our previous experiences with combined human and animal health interventions (17, 32) and in order to capitalise on the added value of the ‘one health’ approach (33).

## Present investigation

### Study population

The study site was located to the southeast of Lake Chad, bordering the lake and the Chari River. The zone extended 100 km from east to west and 50 km from north to south. The study population consisted of mobile pastoralists from three ethnic groups that utilised different livelihoods and husbandry practices. The Foulbé, also called Peul or Fulani, herded the animals close to the riverbanks or open water bodies of Lake Chad. They preferred to graze their animals at the lakeshore, with the animals often standing in the water to feed. In contrast, the Gorane, or Dazagada, generally stayed in drier areas, watering the animals at wells, which capitalised on their considerable well building skills. The third mobile group in the study area was semi-nomadic Arab people, who lived in villages, only moving towards the lake when pasture resources around the villages become depleted towards the end of the dry season.

## Design

The longitudinal study started with 10 camps (4 Foulbé, 3 Gorane and 3 Arab), and the number was increased to 20 camps after 6 months (8 Foulbé, 7 Gorane and 5 Arab), all of which then participated for an additional 12 months.

## Method

Willingness to participate was ascertained with the head (*Boulama*) of each mobile pastoralist camp. After initial agreement, in accordance with cultural norms, the head designated the herd to be included in the study, either his own or that of a son. A camp herd was defined as the animals that were regularly kept together, often including several, closely related households. The camp herds included cattle, goats, sheep, camels, donkeys, horses and chickens. All participating communities were primarily cattle breeding communities. The designated camp herd, along with the households to which the included animals belonged, was enrolled in the cohort. A household was defined as the members who eat and live together. Usually, each wife had her own household, so the husband was counted only in the household of his first wife.

Every 2–4 weeks, a telephone interview was conducted, first with the leader of the camp (or the specific camp herd), who provided information about the herd, and then with his wife who provided information about family members of all included households. It was a priority to involve both men and women in the study in order to get more complementary information. If the wife was unavailable, the entire interview was conducted with the male participant. Interviews were conducted in the local language of each ethnic group or in Chadian Arabic, and the data were then translated into French to complete the questionnaires. After each phone interview, 1,000 FCFA (about US$2) was transferred to the participant's phone as a small compensation for participation. The transfer amount was doubled if the wife also participated in the interview, which was an effective incentive resulting in substantial female participation. The calls were made from a central village in the area, Grédaya, where there is also a weekly market, which is often visited by mobile pastoralists. The interviewer was a local health worker who was well known to the pastoralists. He made an appointment with each participant prior to every phone interview, followed by a reminder a few days prior to the appointment. This approach led to high compliance as the participants made great efforts to attend the interviews, reportedly climbing trees or travelling to nearby places with reliable network coverage.

During each telephone interview, the positions of the camp as well as the distance from the last camp location were orally reported. The narrative description of the localisation was transferred to an oversized Google Earth map (Google Inc., Mountain View, CA, USA) with a scale of 1:25,000 of the study area. The resolution allowed for visual identification of the villages, and positions of camps were localised and geo-referenced. The positions were validated during field visits, when the positions were recorded with GPS devices. The distance between the reported and actual position was calculated using ArcMap 9.2, which was also used for visualisation of the routes (Figs. 6–8).

During the interviews, data were collected about the presence or absence in the camp of each family member, as well as the number of pregnant women, births and deaths. As the communities did not always specifically record the birth dates of babies, we approximated all birth dates to be the middle date between two consecutive interviews.

For the livestock, participants were asked about camp herd structure, births, deaths, sales and purchases of animals, as well as animals taken in or entrusted to other pastoralists. The data on cattle were internally validated and cross-checked between the questionnaires.

Data were collected about cattle, goats, sheep and camels, divided by sex and age group (less than 1 year, 1–2 years and 2 years and older). For cattle, castrated males used for transportation of goods were also counted. We assumed age of first calving of cattle to be 3 years of age (21), which necessitated the inclusion of an adjusting factor to estimate the proportion of females above 3 years of age. Female cattle aged between 1 and 3 years were defined as heifers. As a reference, we used data from Gambia (34). Additionally, data were collected about donkeys, horses (juvenile and adult age groups) and chickens.

The information was recorded on paper questionnaires. Data were double-entered using Microsoft^®^ Access 2002 (Microsoft Corp.; Redmond, USA), compared with Epi Info™ 3.5.1 Data Compare program (Center for Disease Control and Prevention, Atlanta, GA, USA) and cleaned. Statistical analysis was done with Stata IC 10.1 (StataCorp LP, College Station, TX, USA). ArcGIS 9.3 (ESRI Inc. ArcMap™ 9.3, Redlands, CA, USA) and Quantum GIS 1.8.0-Lisboa (OSGeo, Beaverton, OR, USA) were used for geospatial analysis. A demographic cattle model, using cumulated data from the interviews, was generated with Vensim version 6.0 (Ventana Systems, Inc. Harvard, MA, USA). Parameters were optimised on the basis of goodness-of-fit, or ‘payoff’, in Vensim software by comparing the log likelihood of the current model with the log likelihood of a perfect model, with the number of parameters equal to the number of data points. Data on female cows above 3 years of age were adjusted with an adjusting factor, since collected data included all cattle older than 2 years. Birth rates were calculated for each sex and net change rates for each sex and age group.

All members of the participants’ camps who reported illness had access to local health staff during a field visit at least once during the study period. On this occasion, free consultation and examination was provided to all camp members present. Any necessary medications were provided at low cost to the patients. In addition, an emergency service for medical and veterinary problems was established, which continues to date. When a health emergency occurred, participants could telephone a local health worker in Grédaya, who would then travel to their camp. Using a cost sharing system, the participants paid for any medications needed, while the demographic surveillance project paid the transportation cost for the health personnel.

Ethical approval for the study was granted by the Swiss Ethics commission of Basel (EKBB 316/08), and a research permit was also obtained from the Ministry of Health of Chad (No 571/MSP/SE/SG/DGAS/2010).

## Results

### Cohort

A total of 490 telephone interviews were conducted. No enrolled camps were lost to follow-up. The telephone-cohort included 20 camps with one camp herd each and a total of 83 households (range 3–7 per camp). Usually all of the households stayed with the animals, with the exception of one Arab family, where only the herder and his family travelled with the animals throughout the mobile period, while the families of the owners remained in the village. Ten camps were followed for 18 months, participating in 30 interviews each. Six months after the study was initiated, an additional 10 camps were added, which participated in 19 interviews each during the following year.

At the mid-point in November 2011, the small-scale cohort consisted of 579 people, 2,869 cattle, 1,183 goats, 1,198 sheep, 338 donkeys, 99 horses, 35 camels and 315 chickens. All participants complied with the study until closure, resulting in zero dropouts. The mean number of livestock per person was 5.0 cattle (95% CI: 4.0–6.0), 2.0 sheep (95% CI: 0.9–3.1) and 2.1 goats (95% CI: 1.3–3.0).

**Fig. 1:**
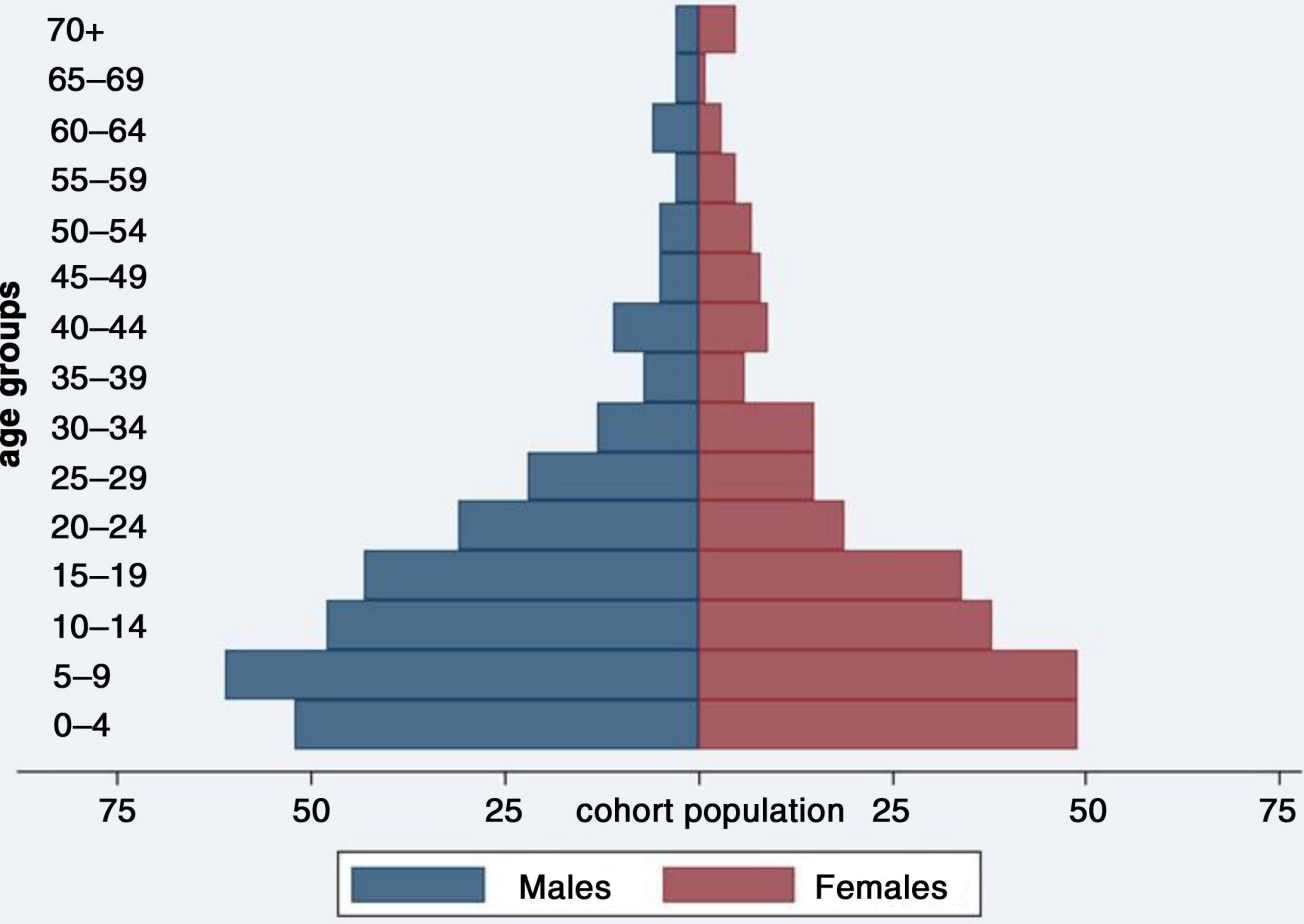
Age distribution of the human cohort in November 2011.

The age distribution of the human cohort is shown in Fig. 1.

One household consisted, on average, of 7.17 household members (SD=2.89) with a minimum of 2 and a maximum of 14 household members. Three households were headed up by women. The number of people per camp ranged from 16 to 55 people belonging to one camp herd of livestock (mean 28.9; SD 9.97).

During the study period, the births of 16 children were recorded. From all reported pregnancies (*n*=24), 42% (*n*=10) did not result in live births, with nine ending in spontaneous abortion and one in stillbirth. Fourteen women had live births, of which two women gave birth to twins. One of the nine miscarriages recorded twin foetuses. The average number of days between first reporting (pregnancies first reported at the beginning of the study period were exempted) and live birth was 152 days (SD: 90, min: 44, max: 251).

During the study period, three deaths were reported: two females aged 10 and 19, respectively, and one male aged 28. The reported cause of death in all cases was disease. Three individuals left the cohort, one due to employment and two due to outside marriage. Two children joined the cohort after temporarily living with extended family in another village. The 16 neonates were added to the cohort.

**Fig. 2:**
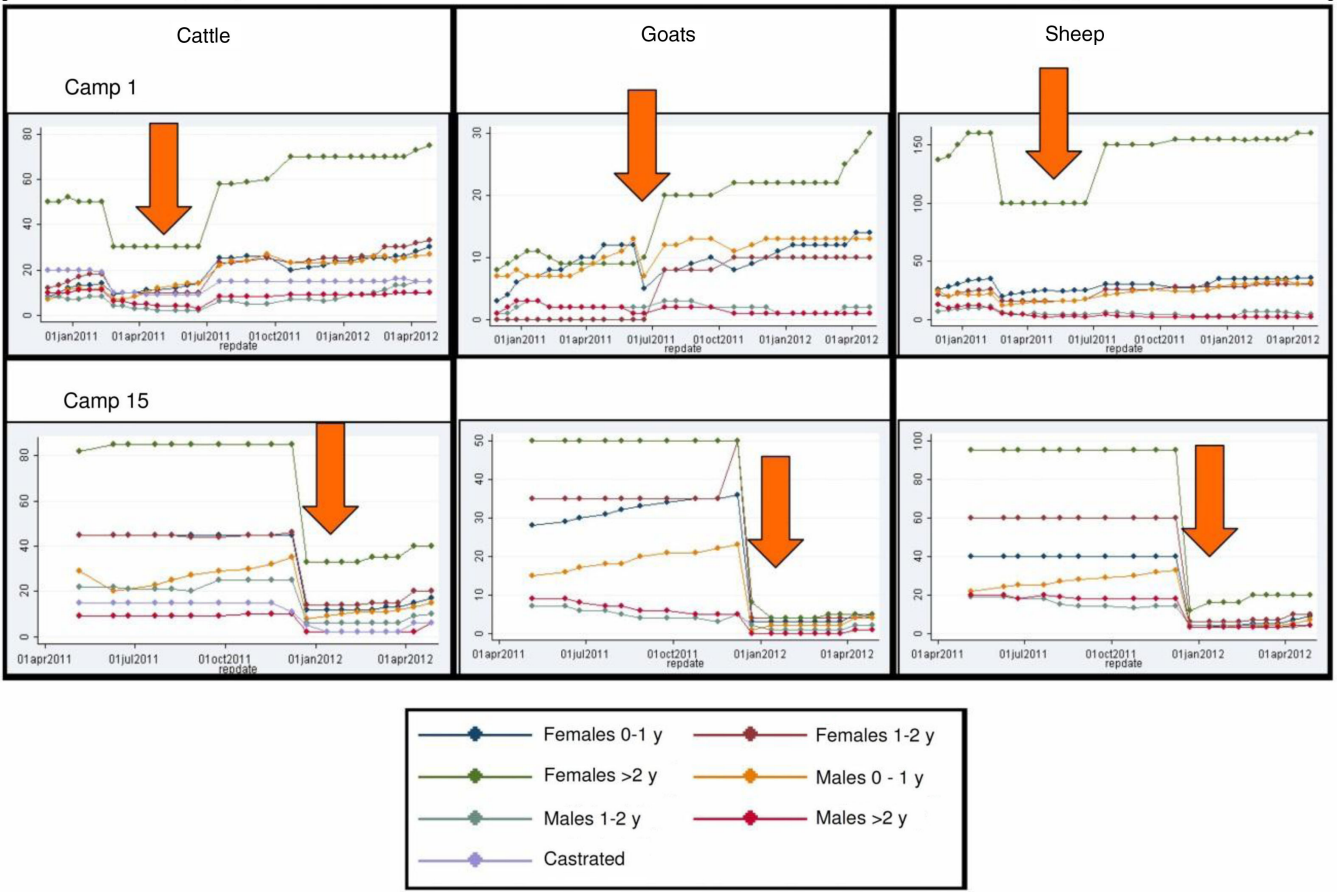
Herd splitting to manage dry season resource scarcity.

In two cases, entire households separated from the others, dividing the herds due to pasture scarcity (Fig. 2), and one household entrusted their animals to others to facilitate working in their fields. During the rainy season, when there was new growth in the pastures and after the harvest was completed, the households reunited.

One wife had, on average, 5.0 living children. Other children in the household were from the extended family, with only one case of an unrelated herder boy residing in a household. There was an average of 3.8 children below 16 years of age in the household, with 1.2 being under 5 years of age. No child attended regular school, although eight boys (between 8 and 23 years) attended Koranic schools in larger villages or towns. Menopausal women (older than 50 years) where the youngest child was still living in the household (*n=*7), had an average of 7.0 living children.

The median animal numbers per herd, including all animal species, are summarised in Table 1.

**Table 1:** Average animal numbers for herds

	Mean	SD	Min.	Max.
Cattle	143.5	66.6	47	258
Goats	59.2	49.0	0	150
Sheep	59.9	78.2	0	259
Camels	1.8	2.3	0	8
Donkeys	16.9	18.3	2	87
Horses	5.0	3.0	1	16
Chicken	15.8	12.0	0	33

Table 2 shows the average number of cattle and small ruminants per herd stratified by age group and sex and the proportion of each group in the species-specific herd.

**Table 2:** Average animal number and proportion of each age group and sex in species-specific herds

	Female animals					Male animals				
		
	Mean	% of herd	SD	Min.	Max.	Mean	% of herd	SD	Min.	Max.
Cattle 0–1	19.9	14	8.3	7	45	18.9	13	6.3	6	32
Cattle 1–2	18.6	13	9.9	5	45	8.3	6	5.8	0	25
Cattle 2+	66.6	46	39.6	20	170	6.2	4	5.7	1	25
Castrated						5.1	4	6.1	0	19
Goats 0–1	12.4	21	10.8	0	35	11.3	19	9.6	0	32
Goats 1–2	11.6	20	10.5	0	35	1.6	3	1.8	0	8
Goats 2+	21.7	37	19.1	0	70	0.7	1	1.2	0	5
Sheep 0–1	10.4	17	12.3	0	40	9.4	16	10.9	0	32
Sheep 1–2	11.1	18	15.0	0	60	1.5	2	3.1	0	14
Sheep 2+	26.1	44	39.0	0	155	1.5	3	4.0	0	18

The fertility rate per year for small ruminants was 1.09 kids per adult female goat (*n=*433) and 0.76 lambs per adult female sheep (*n=*522). Adult ruminants were considered as those aged 2 years and older. For cattle, the cow–calf ratio was 0.68 calves per cow (3 years and older; *n=*1,145). Juveniles (<1 year) comprised 39% of donkeys and 32% of horses.

### Cattle herd development model

**Fig. 3:**
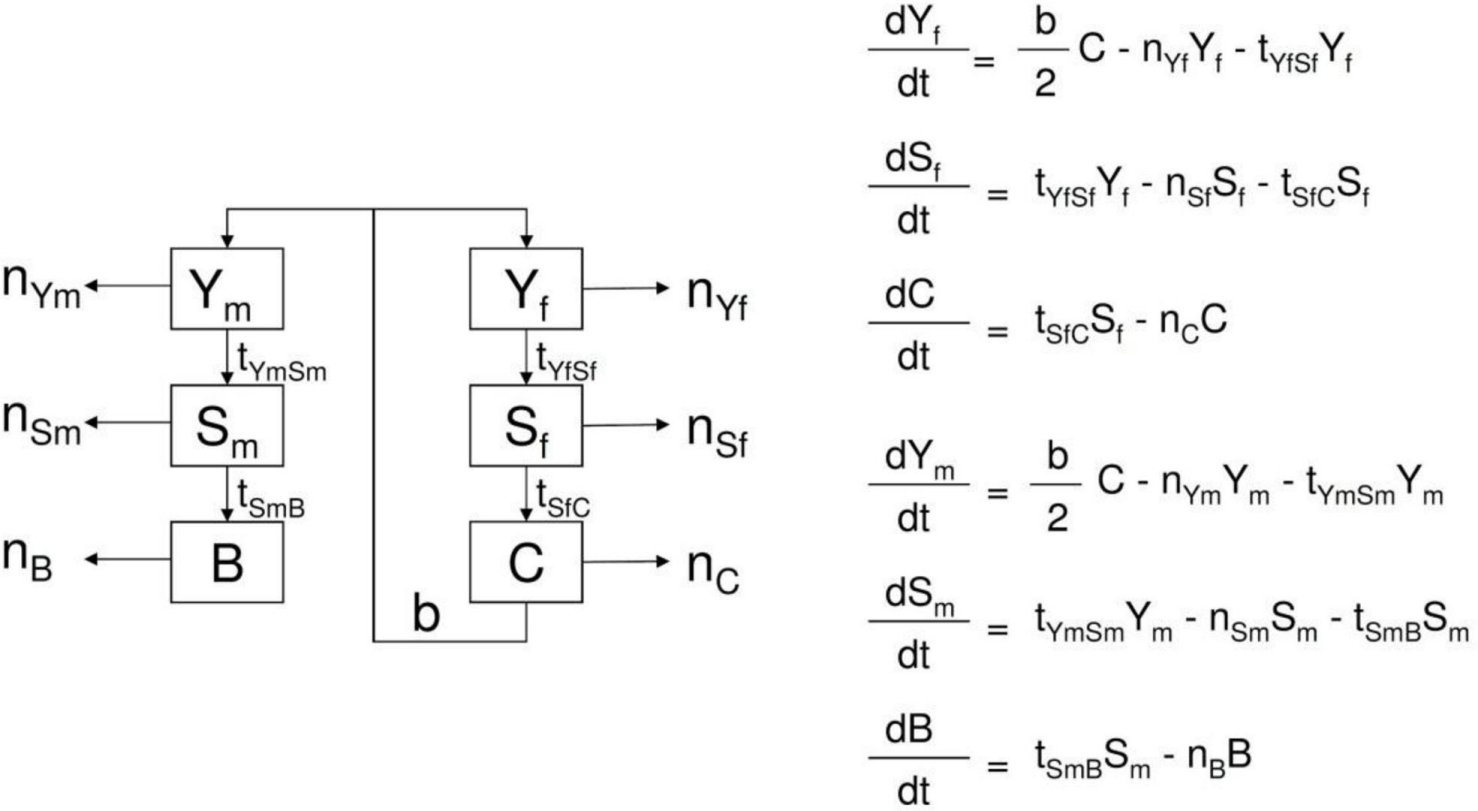
Livestock demographic model and equations, where Yf=young females 0–1 year, Ym=young males 0–1 year, Sf=Heifers 1–3 years, Sm=young bulls 1–2 years, C=cows from 3 years, B=bulls from 2 years, m=mortality (including off-take), b=birth rate (including acquisition), a=age class transition.

**Fig. 4:**
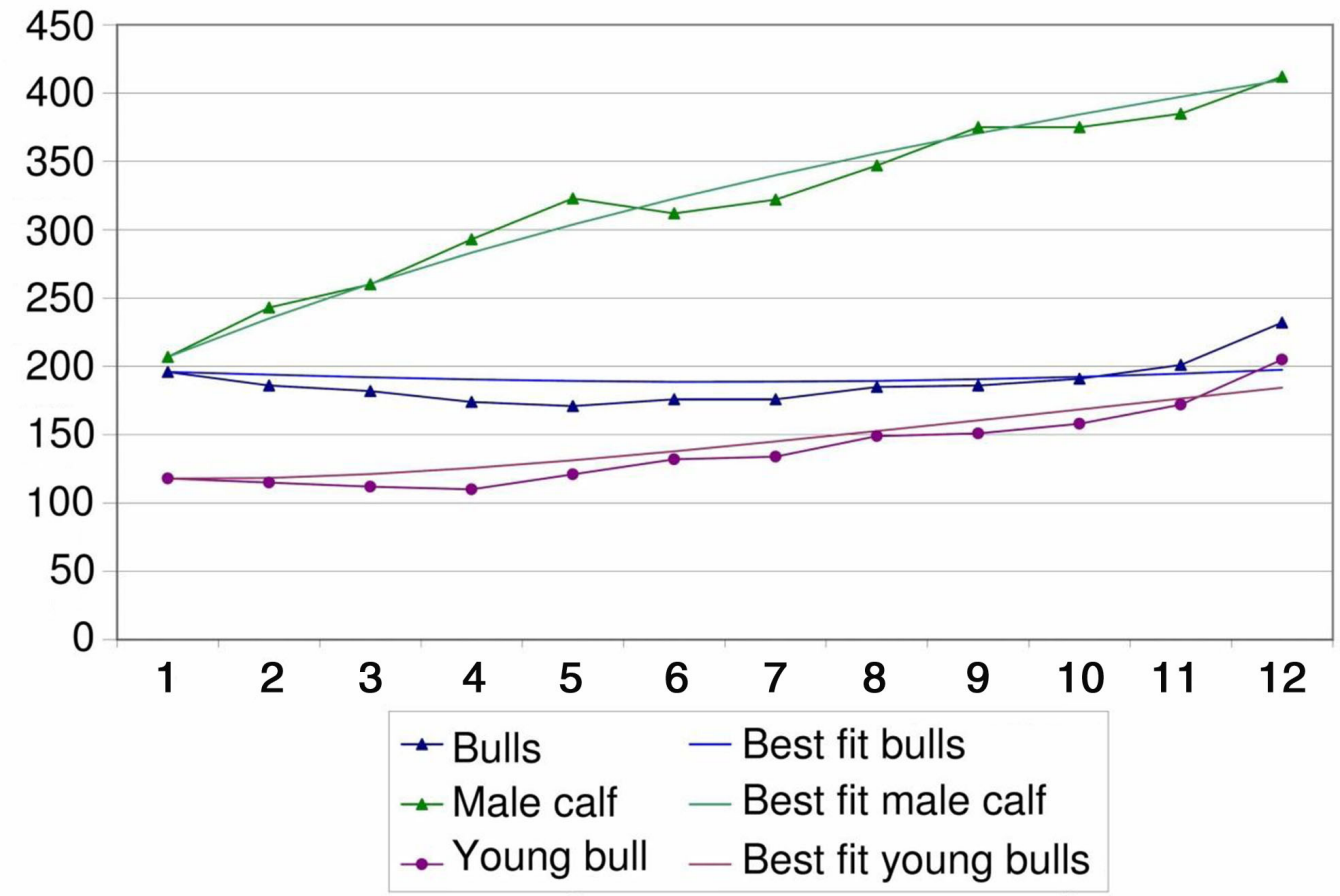
Demographic model fit for male cattle during the time period of 1 year.

**Fig. 5:**
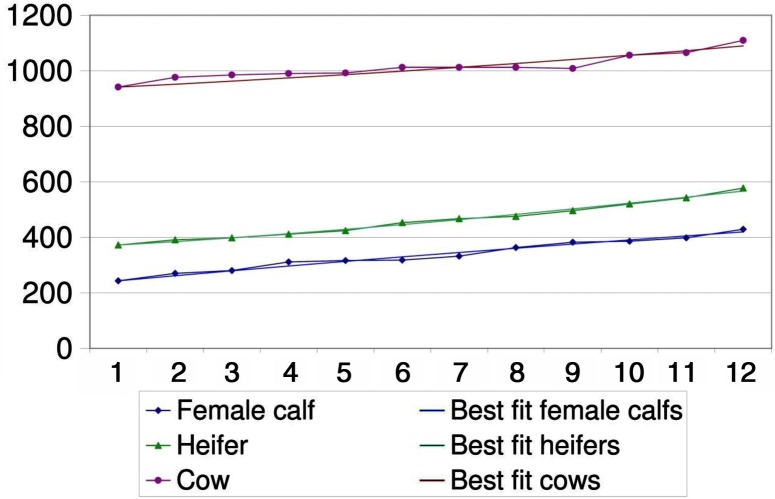
Demographic model fit for female cattle during the time period of 1 year.

The data of all cumulated cattle herds were used to establish the parameters of a herd model shown in Fig. 3. The best fit is shown in Figs. 4 and 5. Parameters are described in Table 3.

**Table 3:** Best-fit parameters for birth rate and mortality per year

	Lower boundary	Best fit	Upper boundary
Birth rates			
Female calves	0.0	0.4	0.5
Male calves	0.6	0.7	0.8
Mortality			
Male calf	0.2	0.5	0.8
Young bull	0.4	0.7	1.1
Bull	0.6	0.7	0.8
Female calf	−0.6	−0.5	−0.1
Heifer	−0.4	−0.2	−0.1
Cow	0.0	0.1	0.1

### Geospatial data of transhumance routes

To validate the oral position descriptions reported by camp leaders, GPS coordinates of 19 camps were recorded, using a Garmin eTrex 10 handheld device, during routine visits for comparison to the reported positions. The average distance between the described and actual positions was 1.79 km (minimum 0.4, maximum 4.0 km).

**Fig. 6:**
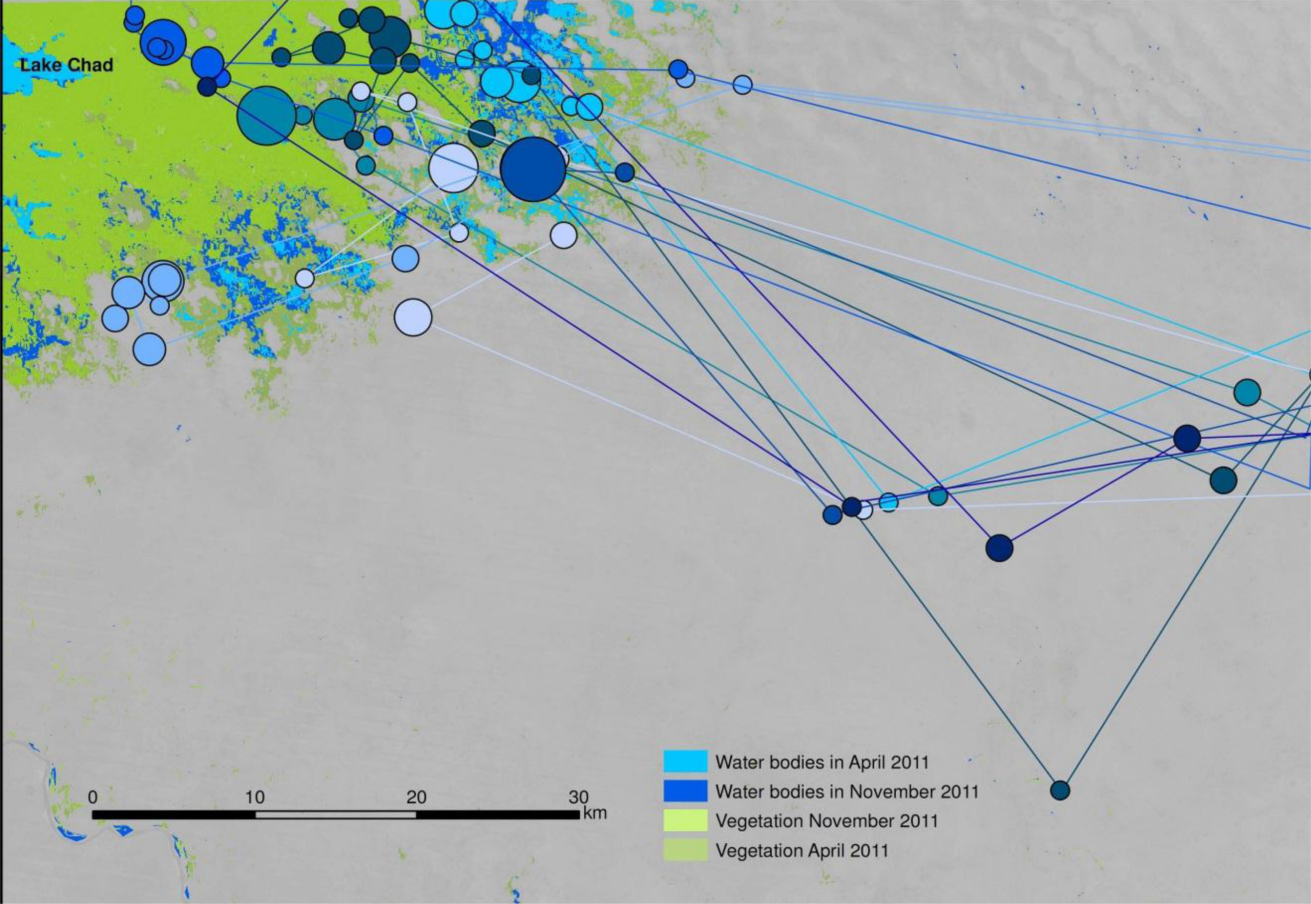
Movement of Foulbé communities in the study zone. Circle size indicates length of stay in each position. Each colour represents a different community.

**Fig. 7:**
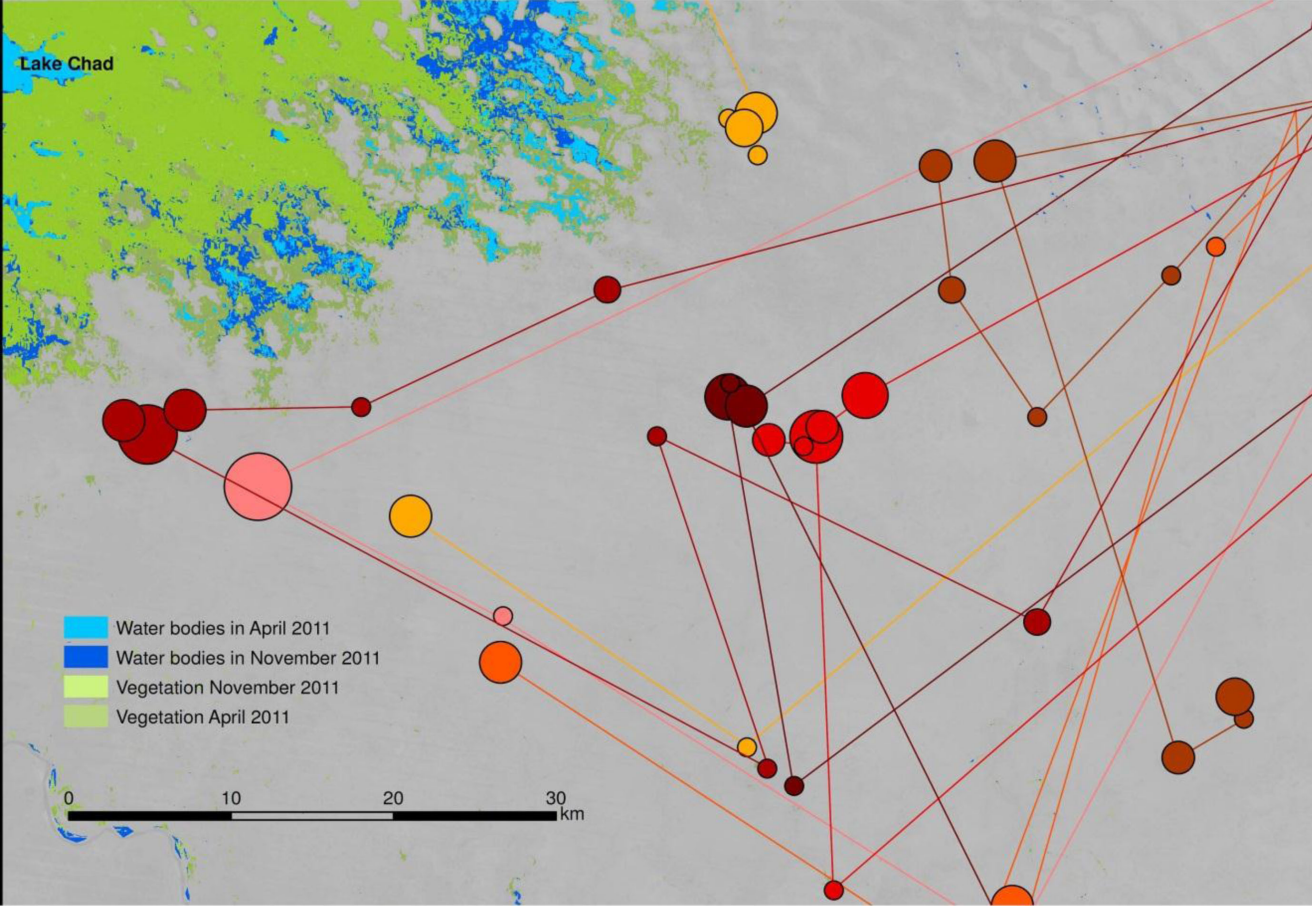
Movement of Gorane communities in the study zone. Circle size indicates length of stay in each position. Each colour represents a different community.

**Fig. 8:**
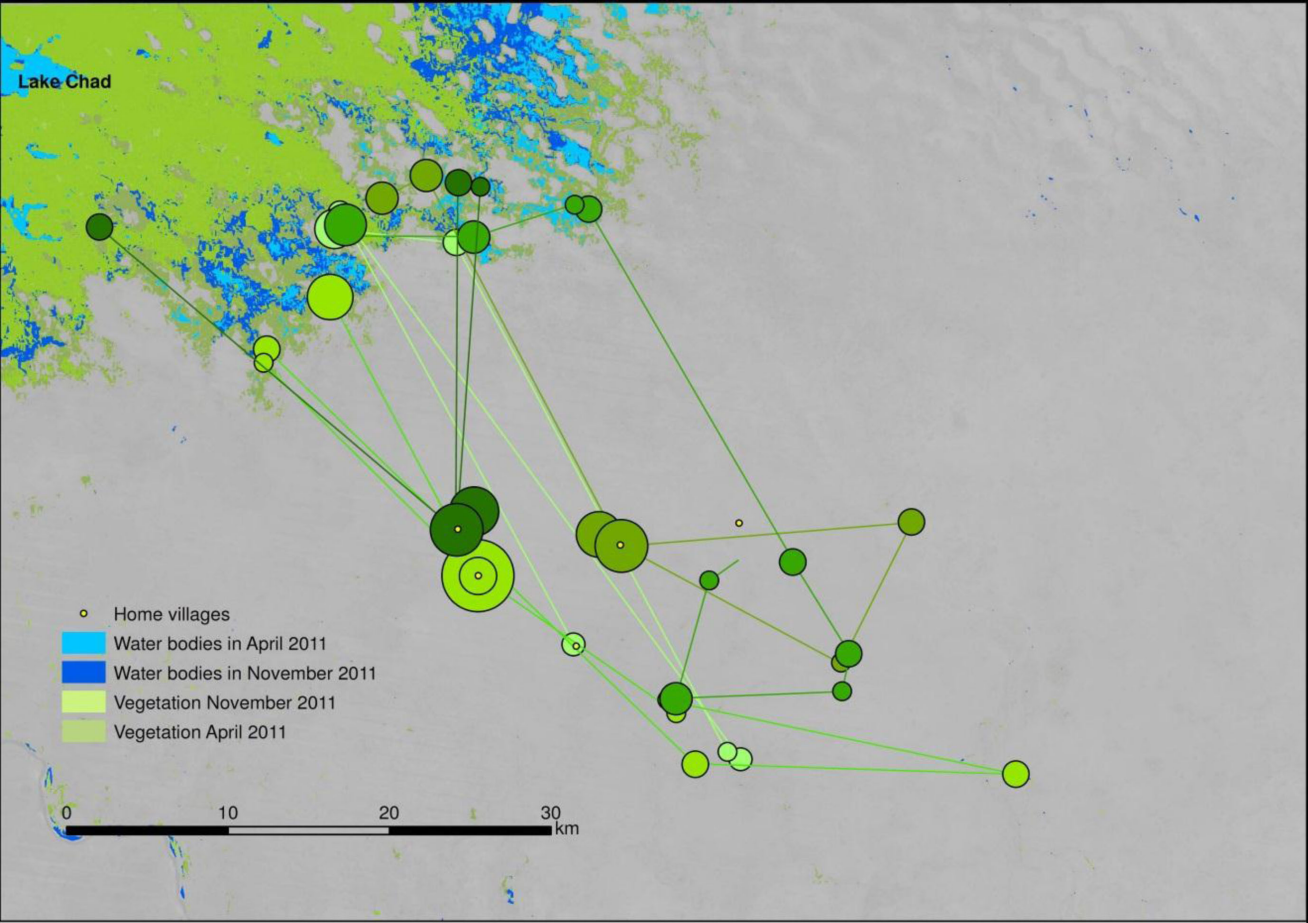
Movement of Arab communities in the study zone. Circle size indicates length of stay in each position. Each colour represents a different community. Yellow points mark position of home villages.

When stratified by ethnic group, a clear pattern of spatial distribution emerges. Foulbé people herd their animals in close proximity to the water bodies of Lake Chad (Fig. 6). In contrast, Gorane people, who are skilled well builders, avoid the shore areas for their camps (Fig. 7). Arab cattle breeders, who are semi-nomadic, remain in their villages as long as there is grass in the surrounding pastures, only moving closer to the lake towards the end of the dry season as resources become scarce (Fig. 8).

### Cost-effectiveness of mobile DSS

Based on the actual cost of the small-scale trial, an estimate of the cost for a full scale mobile DSS was prepared. Assuming an effective surveillance of 20,000 mobile pastoralists and 10,000 sedentary dwellers in the same area of the south-eastern shore of Lake Chad, the cost of surveillance is estimated at 13.2 USD per capita in the first year and 10.5 USD per capita in subsequent years. This would include one phone call per month to each participating family and the cost of operating the call centre. The detailed assessment of costs is published elsewhere (35).

## Discussion

The results of the mobile phone surveillance of mobile pastoralists and their herds clearly show the feasibility and advantages of a mobile DSS. The collection of data provided reliable, nearly real-time information on the human and animal population and their movements. No households were lost to follow-up, even though the study spanned 12–18 months and required participants to be available for regular phone interviews. The data were validated internally through a demographic model for the herds and personal contacts for the human population.

The costs for the small-scale study were low since local staff was engaged using locally available resources. The study population is barely accessible due to the remote location and their mobility. In this regard, a mobile phone system appears to be both adequate and cost-effective. The estimated costs of 13.2 USD per capita for the first year and 10.5 USD per capita for subsequent years would include 12 rounds of follow-up per year, compared to 4.17 USD per capita per year for three rounds of follow-up assumed by INDEPTH sites (36). Even the higher first year cost of the proposed mobile DSS is more cost-effective, especially when considering access for remote mobile and settled populations, the added animal demography aspect and the included emergency health system.

The geospatial analysis reveals different husbandry practices and mobility patterns for the different ethnic groups, confirming the reported disparities of preferential location in close proximity to open water for Foulbé people and in drier areas for Gorane people. It also documents the practise of moving towards Lake Chad for Arab semi-nomadic communities when the pastures around their villages are depleted. These differences are important considerations for future conceptual planning.

Although this study was designed to demonstrate proof of method, rather than to provide demographic results, nonetheless, this information could be used to develop adapted contextual interventions for health and other social services. Information on movements were collected on a close to real-time basis, which provides an opportunity for specific measures and maximises the spatio-temporal accessibility of the camps for social service providers, for instance, aiming to increase coverage of interventions.

The cohort of this study consisted mainly of mobile communities who had previously experienced contact with researchers (17). They were motivated to participate, but this may not always be the case for randomly selected participants of other regions. However, it must be noted that information about this project spread within mobile pastoralist networks, and there was high interest of non-participating camps seeking to participate.

None of the children from the participating households in this study attended a formal school. In explanation, it was often stated that the children are occupied herding the animals. Conflicts between the settled populations and mobile pastoralists are frequently reported (37, 38). The need to accompany herds more closely to prevent crop damage from animals has increased as the number of agricultural fields surrounding water points has expanded (35). Child labour in mobile pastoralist societies is an issue, not only preventing children from receiving formal education, but also causing isolation and exposure to dangerous environments (39). In Chad, an average of only 29% of girls and 47% of boys complete primary school (40). This inequitable access of mobile pastoralists should be urgently addressed by the authorities as well as international and national organisations. Demographic surveillance of these populations could provide missing information for policies and planning with more equitable access.

In this study, pregnancies of women were recorded from an early stage, which allowed for the observation of spontaneous abortions. As the loss of unborn children is still culturally a forbidden topic, it is very difficult to collect reliable information retrospectively and such data collection is associated with much effort and time investment (8). Prospectively with mobile phones, we had valuable insight into the pregnancy outcomes, especially because interviews were conducted with short time intervals. The proportion of pregnancies not resulting in live birth is alarmingly high, although the small sample size has to be considered. This warrants further evaluation within the scale-up of a mobile DSS.

The validation of herd data with effective counts is very difficult due to cultural norms, with a real possibility to disrupt the trust relationship between participants and researchers. A systematic approach to animal health could facilitate animal counts if a certain level of trust was established. Complementary, internal questionnaire validation of herd structures and sizes is proposed to increase the reliability of numbers. Marking the animals of the herd owner could be considered in a further study, but even that does not guarantee the completeness of data.

The demographic model for cattle was established to show the consistency of the reported data as a method of validation. Its outcome indicates a herd in recovery, although the population was too small to provide more specific evidence. Density dependence has not been taken into account to establish the demographic model. The birth rate from the model is comparable to birth rates found previously in African cattle (34). The net change rate represents the natural mortality as well as the off-take and acquisition of animals. From comparable demographic studies, it is assumed that calf mortality in African livestock is between 10 and 20% (below 1 year of age), around 4% for animals between 1 and 2 years and 5% for adult animals (34). The low net change rates for the youngest age group in the demographic model would indicate purchase of young animals. Male animals are often bought for fattening and females for re-stocking. For heifers, the net change rate is negative which indicates substantial acquisition of young females for breeding purposes. This compensates for, and even exceeds, the effect of natural mortality. The same phenomenon was described in Gambia (34). The very high net change rates of bulls, including young bulls, indicate an off-take of animals sold at markets for slaughter.

During the observation period, the animal numbers were clearly increasing. The need for recovery and expansion of herds is consistent with reports from mobile pastoralists of high losses in recent years due to disease and lack of pastures. Nevertheless, the animal density numbers in the area are very high. It is likely that pastoralists apply a form of risk management which maintains high animal numbers, thus exacerbating the problem of decreasing pastoral areas associated with increased small-scale farming. Rising cattle numbers due to altered activities are also mentioned by Wiese in interviews with nomadic pastoralist representatives at local markets (11).

The observation period during this study was quite short and the cattle cohort was relatively small to calculate a solid demographic model for people and animals. More meaningful data should be collected with a long-term full-scale mobile DSS. The demographic data collected on livestock, in addition to the data on humans, shows the potential for a ‘one health’ demographic and health surveillance system for humans and animals. This would clearly have an added value compared to separate surveillance systems with regard to cost and disease information, especially for zoonotic diseases (41).

We recommend expanding this small-scale survey to a cohort of approximately 20,000 participants and their animals. Continued technological developments should allow for GPS tracking of communities and also the use of applications for disease surveillance, even for illiterate people, in the future. Our experiences strongly support the feasibility of a large-scale project which would likely be low-cost, well accepted by the target population and able to provide reliable real-time data. A longer-term mobile demographic health and surveillance system would have benefits in many diverse areas. Health and demographic data could be collected, along with environmental information about rainfall, droughts and locust infestations and economic information such as prices of cereal, milk and livestock. The real-time knowledge on camp locations and populations would facilitate health interventions such as vaccination delivery or sensitisation and information campaigns. Through the emergency medical and veterinary service, an on-going relationship was maintained with the study population. This personal contact built trust between survey staff and participants in addition to enhancing physical validation of telephone interview data. A sustainable emergency health system utilising shared costs, such as the one developed during this study, would be greatly valued by the local populations. Additionally, outbreaks of human and animal diseases, like cholera, measles or anthrax, could be monitored closely, enabling control measures to be quickly implemented. The combined health, ecological and economic information could be processed into an early warning system for humanitarian crisis situations, which occur regularly in the area, thereby facilitating a timely response.

## Conclusion

Mobile phone demographic surveillance of mobile pastoralists and their herds is feasible. This study was limited by its small scale. An extrapolation of the cost per capita for 30,000 fully or partially mobile people in a nearly inaccessible area appears to be more cost-effective than existing DSS (36). A close follow-up of the transhumance patterns could inform health planners on optimal timing and location of preventive interventions to maximise coverage with scarce resources. Near real-time follow-up of pregnancies or other health indicators could allow for the identification of emerging problems more quickly and probably more accurately than through the current official reporting systems. The proposed mobile DSS system could be used for simultaneous health and demographic surveillance of humans and their livestock.

## References

[1] Weibel D, Schelling E, Bonfoh B, Utzinger J, Hattendorf J, Abdoulaye M (2008). Demographic and health surveillance of mobile pastoralists in Chad: integration of biometric fingerprint identification into a geographical information system. Geospat Health.

[2] Sankoh O, Byass P (2012). The INDEPTH network: filling vital gaps in global epidemiology. Int J Epidemiol.

[3] Hill A, Randall S (1984). Différences géographiques et sociales dans la mortalité infantile et juvénile au Mali. Population.

[4] Brainard J (1986). Differential mortality in Turkana agriculturalists and pastoralists. Am J Phys Anthropol.

[5] Roth E (1994). Demographic systems: two east African examples. African Pastoral Systems: an integrated approach.

[6] Leslie PW, Little MA (1999). Turkana herders of the Dry Savana: ecology and biobehavioral response of nomads to an uncertain environment.

[7] Schelling E, Wyss K, Béchir M, Moto DD, Zinsstag J (2005). Synergy between public health and veterinary services to deliver human and animal health interventions in rural low income settings. BMJ.

[8] Münch A (2012). Nomadic women's health practice Islamic belief and medical care among Kel Alhafra Tuareg in Mali.

[9] Thornton P, Kruska R, Henningerl N, Kristjanson P, Reid R, Atieno F (2002). ILRI – mapping poverty and livestock in the developing world. http://www.ilri.cgiar.org/InfoServ/Webpub/fulldocs/Mappoverty/media/.

[10] Rass N (2006). Policies and strategies to address the vulnerability of pastoralists in sub-Saharan Africa.

[11] Wiese M (2006). Health-vulnerability in a complex crisis situation. Implications for providing health care to a nomadic people in Chad. J Afr.

[12] Weibel D, Béchir M, Hattendorf J, Bonfoh B, Zinsstag J, Schelling E (2011). Random demographic household surveys in highly mobile pastoral communities in Chad. Bull World Health Organ.

[13] Schelling E, Diguimbaye C, Daoud S, Nicolet J, Boerlin P, Tanner M (2003). Brucellosis and Q-fever seroprevalences of nomadic pastoralists and their livestock in Chad. Prev Vet Med.

[14] Wyss K, Bechir M, Schelling E, Daugla DM, Zinsstag J (2004). Health care services for nomadic people. Lessons learned from research and implementation activities in Chad]. Médecine Trop Rev Corps Santé Colon.

[15] Wiese M, Donnat M, Wyss K (2004). Health care centre attendance by Arab nomadic pastoralists. A case study in Kanem, Chad]. Médecine Trop Rev Corps Santé Colon.

[16] Kalsbeek WD (1986). Nomad sampling: an analytic study of alternative design strategies. Proceedings of the section on survey research methods. https://www.amstat.org/sections/SRMS/Proceedings/papers/1986_028.pdf.

[17] Schelling E, Bechir M, Ahmed MA, Wyss K, Randolph TF, Zinsstag J (2007). Human and animal vaccination delivery to remote nomadic families, Chad. Emerg Infect Dis.

[18] Zinsstag J, Schelling E, Wyss K, Mahamat MB (2005). Potential of cooperation between human and animal health to strengthen health systems. Lancet.

[19] Bechir M, Schelling E, Wyss K, Daugla DM, Daoud S, Tanner M (2004). [An innovative approach combining human and animal vaccination campaigns in nomadic settings of Chad: experiences and costs]. Médecine Trop Rev Corps Santé Colon.

[20] Hampshire K, Randall S (2000). Pastoralists, agropastoralists and migrants: interactions between fertility and mobility in northern Burkina Faso. Popul Stud.

[21] Homewood K, Randall S (2009). African pastoralist demography. Ecology of African pastoralist societies.

[22] (1993). Survols aériens à basse altitude du cheptel, des habitations humaines et des ressources partorales dans la ‘zone d'oganisation pastorale’, Tchad.

[23] Déglise C, Suggs LS, Odermatt P (2012). SMS for disease control in developing countries: a systematic review of mobile health applications. J Telemed Telecare.

[24] Shet A, de Costa A (2011). India calling: harnessing the promise of mobile phones for HIV healthcare. Trop Med Int Health TM IH.

[25] Lee S, Chib A, Kim J-N (2011). Midwives’ cell phone use and health knowledge in rural communities. J Health Commun.

[26] Robertson C, Sawford K, Daniel SLA, Nelson TA, Stephen C (2010). Mobile phone-based infectious disease surveillance system, Sri Lanka. Emerg Infect Dis.

[27] Thinyane H, Hansen S, Foster G, Wilson L (2010). Using mobile phones for rapid reporting of zoonotic diseases in rural South Africa. Stud Health Technol Inform.

[28] The transformational use of information and communication technologies in Africa. http://siteresources.worldbank.org/EXTINFORMATIONANDCOMMUNICATIONANDTECHNOLOGIES/Resources/282822-1346223280837/MainReport.pdf.

[29] Telecoms overview for Chad, Ethiopia, Guinea, Cote d'Ivoire, Mozambique, Niger, DR Congo (as of 2012) – Africa. http://www.oafrica.com/city-profile/telecoms-overview-for-chad-ethiopia-guinea-cote-divoire-mozambique-niger-dr-congo-as-of-2012/.

[30] Chad – Telecoms, Mobile and Internet – BuddeComm – BuddeComm. http://www.budde.com.au/Research/Chad-Telecoms-Mobile-and-Internet.html.

[31] Wiese M, Yosko I, Donnat M (2004). Participatory mapping as a tool for public health decision-making in nomadic settings. A case study among Dazagada pastoralists of the Bahr-el-Ghazal region in Chad]. Médecine Trop Rev Corps Santé Colon.

[32] Schelling E, Wyss K, Diguimbaye C, Béchir M, Taleb MO, Bonfoh B (2008). Towards integrated and adapted health services for nomadic pastoralists and their animals: a north–south partnership. Handbook of transdisciplinary research.

[33] Zinsstag J, Bonfoh B, Schelling E, Gauthier-Clerc M, Thomas F (2010). Cohérence des systèmes de santé humaine et animale en Afrique: en route pour une santé unique.

[34] Zinsstag J, Ankers P, Dempfle L, Njie M, Kaufmann J, Itty P (1997). Effect of strategic gastrointestinal nematode control on growth of N'Dama cattle in Gambia. Vet Parasitol.

[35] Jean-Richard V (2013). Crowding at Lake Chad: an integrated approach to demographic and health surveillance of mobile pastoralists and their animals.

[36] Ye Y, Wamukoya M, Ezeh A, Emina JBO, Sankoh O (2012). Health and demographic surveillance systems: a step towards full civil registration and vital statistics system in sub-Sahara Africa?. BMC Public Health.

[37] Krönke P, Wyss K, Zinsstag J (2000). Les Principaux Problèmes des Eleveurs Nomades Fulbe Liés à la Santé Humaine Animale.

[38] Bechir M, Schelling E, Bonfoh B (2010). Evolution saisonnière du statut nutritionel des enfants nomades et sédentaires de moins de cinq ans dans le Sahel au Tchad. Med Trop (Mars).

[39] Gooren H (2013). Children's work in the livestock sector: herding and beyond.

[40] The World Bank (2011). Primary completion rate, (% of relevant age group) ∣ Data ∣ Table. http://data.worldbank.org/indicator/SE.PRM.CMPT.FE.ZS?order=wbapi_data_value_2012+wbapi_data_value+wbapi_data_value-last&sort=asc.

[41] Zinsstag J, Meisser A, Schelling E, Bonfoh B, Tanner M (2012). From ‘two medicines’ to ‘one health’ and beyond. Onderstepoort J Vet Res.

